# Unique Circovirus-Like Genome Detected in Pig Feces

**DOI:** 10.1128/genomeA.00251-14

**Published:** 2014-04-10

**Authors:** Andrew K. Cheung, Terry Fei-fan Ng, Kelly M. Lager, David P. Alt, Eric L. Delwart, Roman M. Pogranichniy

**Affiliations:** a Virus and Prion Diseases Research Unit, National Animal Disease Center, USDA, Agricultural Research Service, Ames, Iowa, USA; c Infectious Bacterial Diseases Research Unit, National Animal Disease Center, USDA, Agricultural Research Service, Ames, Iowa, USA; b Blood Systems Research Institute and Department of Laboratory Medicine, University of California at San Francisco, San Francisco, California, USA; d Indiana Animal Disease Diagnostic Laboratory and Department of Comparative Pathobiology, Purdue University, West Lafayette, Indiana, USA

## Abstract

Using a metagenomic approach and molecular cloning methods, we identified, cloned, and sequenced the complete genome of a novel circular DNA virus, porcine stool-associated virus (PoSCV4), from pig feces. Phylogenetic analysis of the deduced replication initiator protein showed that PoSCV4 is most related to a fur seal feces-associated circular DNA virus.

## GENOME ANNOUNCEMENT

In this study, fecal materials collected from swine exhibiting diarrhea between December 2009 and June 2010 were collected by the Indiana Animal Disease Diagnostic Laboratory, Purdue University, West Lafayette, Indiana, and subsequently submitted to the National Animal Disease Center for metagenomic analysis (1). The genome of a circular DNA virus of 2,980 nucleotides (nt), porcine stool-associated virus (PoSCV4) (isolate CP2), was identified. This genome contains two putative major open reading frames (ORFs): ORF1 of 1,155 nt, encoding a putative replication initiator protein (Rep) involved in rolling circle DNA replication, and ORF2 of 1,044 nt, encoding a putative capsid protein (Cap). A BLASTx search of the genome showed that the best hit was to the fur seal feces-associated circular DNA virus (FSfaCV) (GenBank accession no. KF246569) (2). Pairwise comparison of the entire Rep and Cap deduced protein sequences showed that PoSCV4 and FSfaCV share 34% and 48% amino acid identities, respectively. Within the putative Rep, we identified the rolling circle replication motifs I, II, and III (ITAWN, HLOCYFEA, and YCKKEGDF, respectively) and the helicase Walker A and B motifs (GASGSGKS and LWVDEFRG, respectively).

The genome organizations of Pig-CP2 and FSfaCV are similar, both belonging to the type II genome arrangement (3). The two putative ORFs are bidirectionally transcribed and separated by a small intergenic region (SIR) of 378 nt and a large intergenic region (LIR) of 397 nt. Transcription initiates at LIR and terminates at SIR. The LIR contains a stem-loop structure exhibiting a conserved nonanucleotide motif, TAGTATTAC, which is similar to that found in most circoviruses (4).

The data collected indicate that PoSCV4is a novel circular DNA virus (GenBank accession no. KJ417134). Phylogenetically, PoSCV4 and FSfaCV form a unique clade of single-stranded circular viruses.

### Nucleotide sequence accession number.

The complete genome of PoSCV4 (isolate CP2) has been deposited at GenBank under the accession no. KJ417134.
